# Nanobody-based indirect competitive ELISA for the detection of aflatoxin M1 in dairy products

**DOI:** 10.1038/s41598-024-83869-4

**Published:** 2025-01-04

**Authors:** Li Yi, Haiyuan Liu, Yingda Liu, Aiyisi, Jing He, Liang Ming, Jirimutu

**Affiliations:** 1 Key Laboratory of Dairy Biotechnology and Engineering, Ministry of Education, College of Food Science and Engineering, Inner Mongolia Agricultural University, Hohhot, 010018 China; 2 Inner Mongolia Autonomous Region International Mongolian Hospital, Hohhot, 010018 China

**Keywords:** Aflatoxin M1, Indirect competitive ELISA, Nanobody, Dairy products, Biological techniques, Biophysics

## Abstract

Aflatoxin M1 (AFM1) is known to be carcinogenic, mutagenic, and teratogenic and poses a serious threat to food safety and human health, which makes its surveillance critical. In this study, an indirect competitive ELISA (icELISA) based on a nanobody (Nb M4) was developed for the sensitive and rapid detection of AFM1 in dairy products. In our previous work, Nb M4 was screened from a Bactrian-camel-immunized phage-displayed library. It exhibits VH-like features, possesses higher thermal stability than monoclonal antibody (mAb 1E6) and tightly binds to AFM1–BSA with a *K*_D_ value of 2.5 nM. Under the optimal conditions, its half-maximal inhibitory concentration was 0.338 ng/mL, the limit of detection was 0.051 ng/mL, and linearity was noted in the range of 0.168–0.679 ng/mL. Nb M4 displayed almost no cross-reactivity with other mycotoxins. No matrix effect was observed in milk and milk powder samples, and the matrix effect in yogurt samples could be weakened by 2-fold dilution. Furthermore, validation studies in spiked samples (milk, yogurt, and milk powder) resulted in good recoveries of 95.40–111.33%, with a low coefficient of variation (2.89–6.78%). High-performance liquid chromatography was used to evaluate the accuracy and reliability of the developed icELISA, which indicated a satisfactory consistent correlation (R^2^ = 0.9722). This study highlights the potential of Nb M4 as a promising component for detecting AFM1 in dairy products.

**Supplementary Information:**

The online version contains supplementary material available at 10.1038/s41598-024-83869-4.

## Introduction

Mycotoxin contamination affects approximately 60–80% of food crops globally, potentially leading to “mycotoxicosis” when the contaminated food is consumed by humans as components in livestock feed^1,2^. Aflatoxins, which are one of the most hazardous mycotoxins, are toxic secondary metabolites produced by certain *Aspergillus* spp. under appropriate conditions in various agricultural and food products^3–6^. These toxins cause health and economic issues affecting consumers and farmers worldwide. Approximately 20 AF types have been identified so far, of which aflatoxin B1 (AFB1) is the most potent. AFB1 is carcinogenic, genotoxic, mutagenic, and teratogenic and is categorized as a group 1 carcinogen in the International Agency for Research on Cancer (IARC) classification of carcinogenic substances^7–10^. When animals consume AFB1–contaminated feed, the toxin is metabolized to monohydroxy derivate AFM1 in their liver. This reaction is catalyzed by the hepatic cytochrome P 450 (CYP450) enzyme system, and AFM1 is then eliminated in blood, milk, tissues, and biological fluids^11–14^. As AFM1 binds to milk proteins, especially casein, it is often detected in dairy products (e.g., milk, yogurt, cheese, butter, and infant formula)^15–17^. Approximately 0.3–6.2% of AFB1 is converted into AFM1, but the extent of transformation depends on the diet type, ingestion and digestion rates, animal health, hepatic biotransformation capacity, and milk yield^18,19^. Several in vitro experiments have established that AFM1 is cytotoxic to human hepatocytes and results in hepatocyte degeneration, necrosis, cirrhosis, etc. Moreover, AFM1 is closely linked to the occurrence of various cancers, such as hepatocellular carcinoma, breast cancer, and colon cancer^20,21^. Furthermore, AFM1 may cause DNA damage and mutation, resulting in abnormal cell growth and function as well as negatively impacting gene stability and genetic information transmission^22^. AFM1 is less hazardous than AFB1; nevertheless, it can cause growth suppression, immunological problems, and cancer in both humans and animals^23^. Therefore, AFM1 is also classified as a group I carcinogen by IARC^24^.

Milk and dairy products are excellent sources of fat, protein, and trace elements for humans and are often added to various processed foods as a technical aid^25^. Unfortunately, owing to the high thermal stability of AFM1, it is not degraded or destroyed during sterilization, storage, and processing^26,27^. Hence, this toxin undoubtedly poses severe health risks for consumers, especially in immunologically vulnerable age groups. To avert the adverse health impacts caused by AFM1, many countries have set the maximum residue level (MRL) for AFM1 in dairy products. In the European Union, the concentration of AFM1 in raw milk, heat-treated milk, and milk for the manufacture of dairy products should not exceed 0.05 µg/kg for adult consumption, and 0.025 µg/kg for infants and young children^28^. In the United States and several Asian countries (including China), the permissible limit for AFM1 residues in dairy products and milk-containing foods has been set at 0.5 µg/kg^11,12,29^. These strict limits make AFM1 surveillance critical. The current gold standard methods for AFM1 detection are instrumental methods, such as high-performance liquid chromatography (HPLC) and liquid chromatography–tandem mass spectroscopy (LC-MS/MS)^30,31^. Although these methods are sensitive, accurate, and reliable, they require expensive equipment, extensive sample pretreatment, and specialized personnel. Hence, developing a rapid, sensitive, and accurate method for monitoring AFM1 levels in milk and dairy products is of immense significance.

The enzyme-linked immunosorbent immunoassay (ELISA) is based on antigen–antibody reactions and is commonly used in food industries and official food control agencies. This technique offers high selectivity and sensitivity, requires a small sample volume, is cost-effective, has a wide dynamic measuring range, and enables high-throughput parallel sample processing. Moreover, ELISA offers the advantages of simplicity of operation, and portability^32^. Over the past years, several ELISA-based methods have been introduced to detect AFM1 in milk and dairy products, most of which are competitive methods based on monoclonal antibodies (mAbs)^33–36^. The use of selective antibodies that do not cross–react with other comparable compounds is crucial for the immunoassay detection of tiny molecules^37^. However, mAbs have certain limitations, such as slow and expensive manufacturing and insufficient organic solvent stability to withstand the high concentrations used to extract the targeted toxin causing food contamination^38^. Nanobodies (Nbs), also referred to as Single–domain antibodies (SdAbs), are the variable domains of a heavy-chain antibody (VHH) obtained from Camelidae species and sharks. Nbs are the smallest available antibody fragment with functional antigen binding^39^. Compared to conventional antibodies, Nbs are characterized by small size (approximately 15 kDa); high affinity, selectivity, solubility, and yield; low-cost generation, and increased temperature and organic–solvent stability^40,41^. These advantages make Nbs ideal reagents for the detection of small molecule contaminants^42,43^.

In our previous study, a high-quality phage library was generated via the immunizations of a Bactrian camel with AFM1–BSA. Subsequently, using AFM1–OVA as the screening antigen, acidic elution, and competitive panning strategies were screened to obtain six AFM1–specific nanobodies named Nb M1-M6^44^. This research aimed to express these six Nbs to screen the candidate whose IC_50_ met the detection criteria and evaluate its thermal stability and affinity. Furthermore, an indirect competitive enzyme-linked immunosorbent assay (icELISA) was developed as a potential tool for accurate, sensitive, and selective detection of AFM1 in dairy products.

## Materials and methods

### Materials and reagents

Six AFM1–specific plasmids (pComb3XSS-Nb-TG1) were obtained from our previous study^44^. AFM1 was purchased from Sigma (St. Louis, MO, USA) and AFM1–BSA from Immunechem (Burnaby, British Columbia, Canada). The rat anti-AFM1 monoclonal antibody 1E6 (mAb 1E6) was purchased from Abnova (Taipei, China). AFM2, AFB1, AFB2, AFG1, AFG2, zearalenone (ZEN), deocynivalenol (DON) standards and AFM1 immunoaffinity column were sourced from Pribolab (Qingdao, China). HRP-conjugated anti-6×His-tag monoclonal antibody (anti-His-tag HRP), and helper phage M13KO7 were procured from Nbbiolab (Chengdu, China). *E. coli* Top10F’ competent cells were from Yeasen (Shanghai, China). Ampicillin, kanamycin, neomycin sulfate, agar, 3,3,5,5-tetramethylbenzidine (TMB), tween-20, and skim milk were purchased from Solarbio (Beijing, China). Isopropyl β-D-1-thiogalactoside (IPTG) and bovine serum albumin (BSA) were obtained from Sigma (MO, USA). Qiagen plasmid miniprep kit was purchased from Qiagen (Dusseldorf, Germany). The xTractor cell lysis buffer was sourced from Takara (Shiga, Japan). Costar-96 well EIA/RIA plates were procured from Corning Inc. (Corning, NY, USA). All other organic solvents and inorganic chemicals were commercially available and of reagent grade.

### Methods

#### Production, purification, and characterization of recombinant nbs

Six AFM1-specific plasmids were transferred into *E.coli* Top10F’ competent cells via the heat shock method. The Nbs were expression and purification methods of Cai^45^ reported previously were used in this study. Briefly, pComb3XSS-Nb (M1-M6) -Top10F’ was inoculated in 500 mL SB-Amp culture at a ratio of 1:100 and incubated at 37 °C and 250 rpm until the OD_600_ reached 0.8–1.0. Subsequently, 1 mM IPTG was added into the culture and incubated at 37 °C with shaking at 250 rpm overnight. The supernatant was then centrifuged at 4 ℃ and 5000 g for 10 min and discarded. The periplasmic proteins were isolated using the xTractor cell lysis buffer protein extraction reagent according to the manufacturer’s instructions. After centrifugation, the soluble Nbs in the supernatant were purified using the Ni-NTA resin. Finally, the purified Nbs were dialyzed overnight in the 0.01 M phosphate-buffered saline at pH 7.4 (1× PBS) and characterized by 12% sodium dodecyl sulfate-polyacrylamide gel electrophoresis (SDS-PAGE). The concentration was determined by Nanodrop and stored at − 20 ℃ until further analysis.

#### Binding affinity

The binding affinity of Nb M4 to the AFM1-BSA conjugate was determined with Bio-Layer Interferometry (BLI) using the OctetRED96e System at 25 ℃. (FortéBio, CA, USA). The Ni-NTA biosensors were incubated in 200 µL of kinetic buffer (1× PBS with 0.02% Tween-20, pH 7.0) for 600 s. The His-tagged protein Nb M4 was immobilized at a concentration of 2 µg/mL on Ni-NTA biosensors for 120 s before baseline equilibration for 90 s in kinetic buffer. Later, a two-fold dilution series of the AFM1-BSA conjugate (12.5–100 nM) was dipped in the sensors for 120 s (association step), followed by 180 s dissociation time in the same buffer. The data were baseline-subtracted before being fitted using a 1:1 binding model and the Octet Data Analysis software v10.0 (FortéBio). The equilibrium dissociation constant (*K*_D_) is calculated using the formula *K*_D_ = rate constants of dissociation (*K*_off_)/ rate constants of association (*K*_on_).

#### Thermal stability

The thermal stability of the antibody is a key factor that influences the performance of immunoassays. To compare the thermal stabilities of Nb M4 and mAb 1E6, they were heated at different temperatures for the same duration and at the same temperature for different periods of time using a polymerase chain reaction (PCR) unit. Nb M4 and mAb 1E6 were diluted with 1× PBS to the experimental concentration of 0.1 µg/mL and 1 µg/mL, respectively. They were heated at different temperatures (4, 25, 37, 50, 60, 70, 80, and 90 ℃) for 10 min, respectively. The temperature was then adjusted to 37 ℃ and 85 ℃ for 10, 20, 30, 40, 50 and 60 min. The antibodies were then cooled to room temperature, and their binding activity to coating antigen AFM1–BSA (2 µg/mL) was tested by indirect ELISA (iELISA) and compared with the original activity. The relative activity was calculated using the following formula: relative activity (%) = [OD_450 (the treatment activity)_/ OD_450 (the original activity)_]×100. In addition, the differential scanning calorimetry (DSC) of Nb M4 and mAb 1E6 prepared in 1× PBS (0.5 mg/mL) was performed on the DCS-60 A instrument (Shimadzu, Japan). The thermal scans were conducted from 20 ℃ to 90 ℃ at the rate of 1 ℃/min. The data were collected and analyzed using the TA Instruments NanoAnalyze™ software v3.12.0.

#### Sensitivities of icELISA based on Nbs

A microplate was coated with 100 µL of 2 µg/mL AFM1-BSA in 0.05 M carbonate buffer solution pH 9.6 (CBS) and incubated at 4 ℃ overnight. After three washings, 300 µL of 5% skim milk in 1× PBS with 0.1% Tween 20 was added to the block at 37 ℃ for 1 h. After three washings, 50 µL of two-times titer dilution of Nbs was added to the wells with an equal volume of serial concentrations of AFM1 standard and incubated at 37 ℃ for 1 h. After four washings, 100 µL of anti-His tag HRP monoclonal antibody (1:10000 in 5% skim milk) was added to the wells and incubated at 37 ℃ for 1 h. After five washings, 100 µL of TMB solution was incubated for 7 min and the enzymatic reaction was stopped with 50µL of 1 M H_2_SO_4_. The absorbance at 450 nm was measured with a Multiskan FC Microplate photometer (ThermoFisher, NY, USA). The standard curve was fitted with the four-parameter fitting module of Origin v9.5 (Origein Lab Crop, Northampton, MA, USA). The value of “B/B0” was characterized by the binding ability of Nbs to the coating antigen on the microplate, where B and B0 represented the binding abilities of the Nbs in the presence and absence of AFM1-BSA, respectively. The half-maximal inhibitory concentration (IC_50_) was defined as the concentration of AFM1-BSA that resulted in an inhibition rate of 50%.

#### Optimization of icELISA

To improve the sensitivity of icELISA, a series of experimental parameters that may affect the assay performance were optimized. The binding specificity of Nb M4 to the binding site of the AFM1-BSA coating conjugate was initially optimized using the checkerboard titration method. The coating antigen AFM1-BSA conjugate was diluted to 0.5, 1, 2, and 4 µg/mL in 0.05 M CBS pH 9.6, and the 1 mg/mL Nb M4 was two-fold diluted to serial concentrations (1:1000–1:32000) in 0.5% skim milk (in 1× PBS with 0.1% Tween-20). Other parameters were evaluated including blocking buffers (5% skim milk, 5% BSA, and 5% OVA), blocking time (1 h and 2 h), pH(5.0, 6.0, 7.0, 7.4, 8.0, and 9.0), ionic strength (5, 10, 20, 40, 60, and 80 mmol/L of PBS) and methanol content (5%, 10%, 20%, 40%, 60%, and 80%), which were optimized to enhance the sensitivity of the icELISA. The icELISA procedures were performed as in Sect. 2.2.4 described above. The IC_50_, the limit of detection (LOD, IC_10_), and the linear range (IC_20_–IC_80_) were calculated from the standard curve.

#### Cross-reactivity

To evaluate the specificity of the developed icELISA, the cross-reactivity (CR) of Nb M4 with a group of mycotoxins (including AFM2, AFB1, AFB2, AFG1, AFG2, DON, and ZEN) was determined. In brief, a series of concentrations of these mycotoxin standards (0, 3.125, 6.25, 12.5, 25, 50, and 100ng/mL) were added to the wells in place of the AFM1 standard solution, and standard competitive inhibition curves were plotted for each mycotoxin to calculate the cross reactivity. The cross-reactivity was calculated using the formula as follows: CR (%) = (IC_50_ of AFM1/IC_50_ of other mycotoxins) ×100.

#### Assay validation

The accuracy and precision of the icELISA were determined using the spike-and-recovery test. The milk, yogurt, and milk powder samples were purchased from a local market (Hohhot, China) and were verified as AFM1-free with HPLC analysis. For this assay, 10 g of milk, yogurt, and milk powder solution (0.01 g/L in water) were weighed and spiked with three different concentrations of AFM1 (0.4, 0.6, and 0.8 ng/mL)^46^. All samples were centrifuged at 4 ℃ and 6000 rpm for 10 min, the fat layer was discarded and then analyzed as described above.

The HPLC validation was carried out according to GB5009.24-2016^47,48^. Briefly, 4 g of milk, yogurt, and milk powder solution were mixed with 10 mL ethanol, vortexed for 3 min, and centrifuged at 4 ℃ and 6000 rpm for 10 min. The supernatant was mixed with 1× PBS at a ratio of 1:8, loaded onto an AFM1 immunoaffinity column (Pribolab, Qingdao, China), equilibrated with 2 × 10 mL ultrapure water twice, and then eluted with 2 mL of methanol. The eluate was evaporated to dryness under nitrogen at 50 ℃ and dissolved in methanol. The solution was then filtered with a 0.22 μm membrane for HPLC (SHIMADZU, Japan). The mobile phase comprised water: methanol: acetonitrile at a ratio of 70:15:15 (v/v) with a flow rate of 1 mL/min and loaded with an aliquot of 10 µL filtered samples. The column temperature was maintained at 40 ℃. The excitation and emission wavelengths were set to 360 nm and 430 nm, respectively.

## Results and discussion

### Recombinant production, purification, and characterization of Nbs

**Fig. 1:**
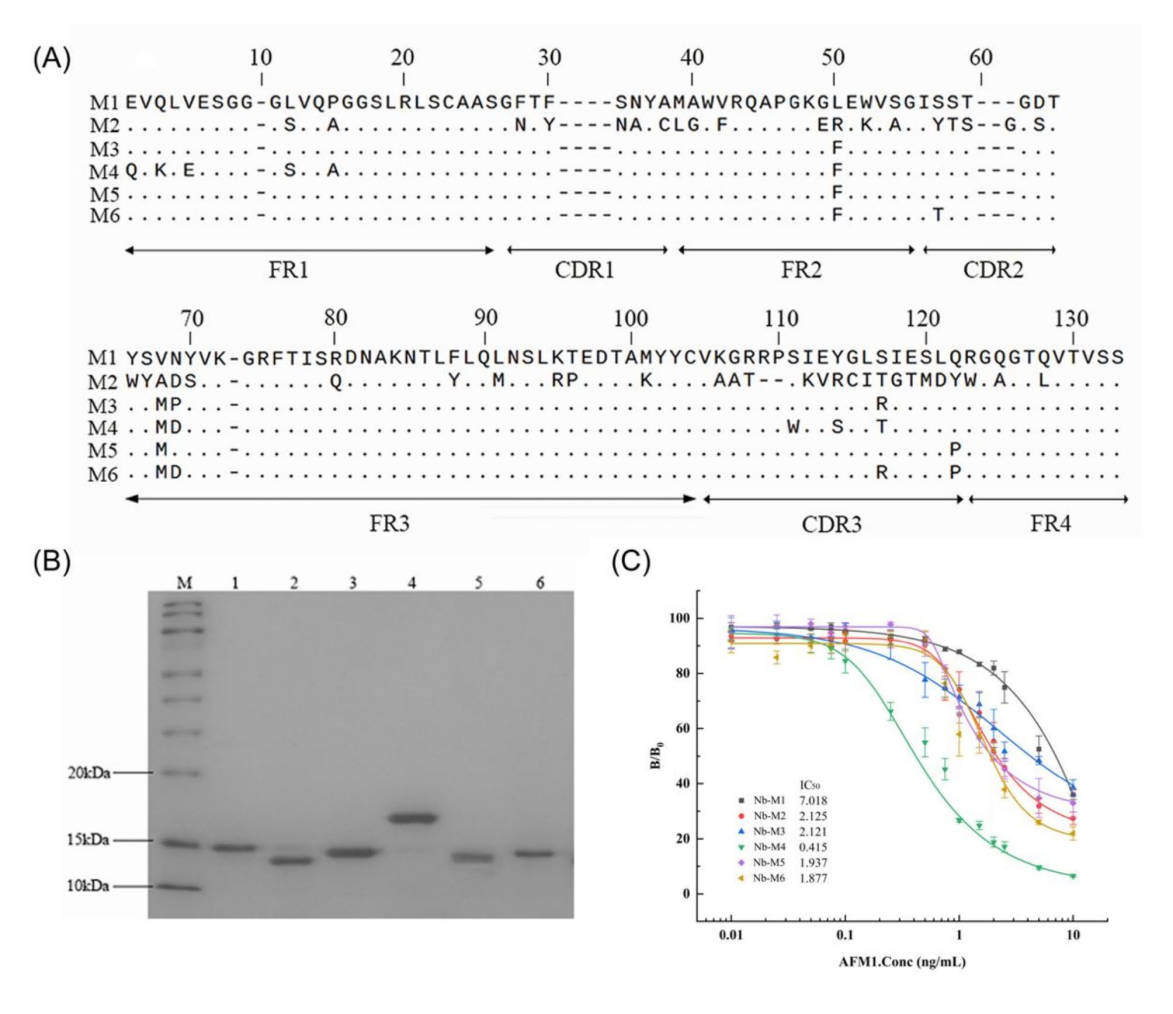
Characterization of anti-AFM1 nanobodies isolated from phage library. (**A**) Amino acid sequence alignment of the six selected Nbs (Nb M1–M6, numbering according to IMGT). The position 10 in the FR1-IMGT and position 73 in the FR3-IMGT are gaps introduced to align to other V-GENE groups or subgroups. Frameworks 1 to 4 and CDRs 1 to 3 were indicated. (**B**) SDS-PAGE analysis of the purified Nbs on 12% gel. M: Marker, lanes 1–6: Nb M1–M6, respectively. (**C**) The competitive inhibition curves of the six Nbs. Representative curves showing the inhibition of each Nb (at two-tomes titer dilution) binding to AFM1–BSA with increasing concentration of free AFM1 (0-10ng/mL). The data represent the mean ± standard deviation (SD) of 3 independent experiments performed in triplicate.

Six AFM1-specific Nbs (M1-M6) plasmids with unique sequences were transformed into the nonsuppressor *E.coli* Top10F’ for expression. The His-tagged Nbs were purified using immobilized ion metal affinity chromatography (IMAC). As illustrated in Fig. 1A, the framework regions (FRs) are highly conserved among six Nbs’ sequences, but with more variation on CDR3. Except Nb M2, the remaining Nbs lack the hallmark hydrophilic amino acid substitutions at positions V42, G49, L50, and W52 in the FR2 region. The IGHV germline genes exhibit high conservation of V42, G49, L50, and W52 across vertebrates, specifically mammals, owing to their interaction with the VL domain. In contrast, the VHH domain does not require pairing with the VL domain, which allows greater flexibility in these amino acids. These amino acids will exhibit greater variability in VHH sequences, particularly after somatic hypermutation events^49^. Moreover, Nb M2 has an additional disulfide bond between CDR1 and CDR3, which could increase the stability due to its lower energy bond with negative sign patterns^50^. This finding might suggest that most of the Nbs we obtained were of the VH-like type. To our knowledge, this is the first report of VH-like Nbs specifically binding to the AFM1 antigen. Subsequently, the expression of Nbs was identified by 12% SDS-PAGE gels with molecular masses ~ 15 kDa, except for Nb M4 (Fig. 1B). The observed difference in Nb M4 was attributed to its C-terminus linkage to the hinge region sequence, which resulted in a theoretical molecular weight of 17.4 kDa (including the 6× His-tag). Generally, the CDR regions of the nanobodies are primarily responsible for antigen recognition^51^, while the hinge region located at the C-terminus provides sufficient sustained the spacing for the antigen-binding sites without significantly affecting their function^52^. Furthermore, the sensitivity of each Nb was determined using icELISA, and the competition inhibition curves were plotted. Nb M4 demonstrated the highest sensitivity, with an IC_50_ of 0.415 ng/mL and a linear range of 0.141–1.652ng/mL (Fig. 1C), which complies with the detection limit of AFM1 in dairy products in several countries. Therefore, Nb M4 was employed for further investigation.

### Thermal stability

**Fig. 2:**
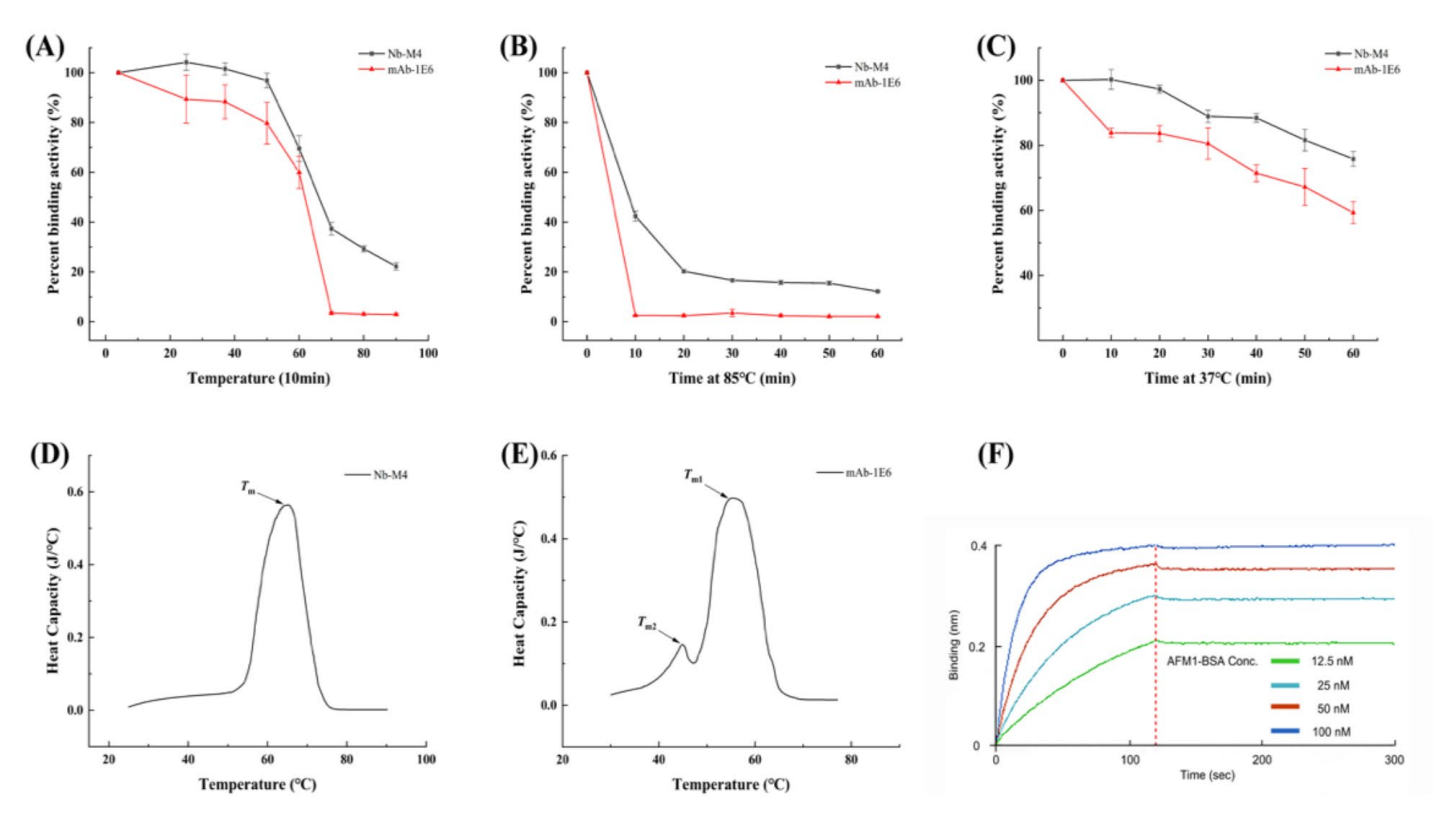
Comparison of the thermal stabilities of Nb M4 and Anti-AFM1 monoclonal antibody (mAb 1Eb). (**A**) The antibody activity of Nb M4 and mAb 1E6 incubated at different temperatures for 5 min. (**B**) The antibody activity of Nb M4 and mAb 1E6 incubated at 37 ℃ for 0–60 min, respectively. (**C**) The antibody activity of Nb M4 and mAb 1E6 incubated at 85 ℃ for 0–60 min, respectively. After cooling to RT, their binding activity to coated 2 µg/mL AFM1–BSA in 96 well microtiter plate was measured by indirect ELISA. (**D**,**E**) Analysis of the apparent melting temperature of Nb M4 and mAb 1E6. (**F**) The Nb M4 was immobilized on an Ni-NTA bio-sensor and the binding kinetics were monitored by bio-layer interferometry (BLI) on OctetRED96 (ForteBio). The biosensor fixed the concentration of the Nb M4 at 2 µg/mL, and the antigen AFM1–BSA concentration was doubled from 12.5nM to 100nM. The measured responses were fitted to a monophasic 1:1 binding model. All values are the mean ± SD of triplicates.

The thermal stability of Nbs plays a pertinent role in various application-related parameters, such as conformational stability, expression yield, and shelf life^45,53,54^. In this study, the thermal stability of Nb M4 was confirmed using the anti-AFM1 mAb (1E6) as a control. The results indicated that Nb M4 maintained nearly 20% of its activity after heating at 90 °C for 10 min, whereas mAb 1E6 completely lost its binding ability when heated at 70 °C for 10 min (Fig. 2A). The results of heating at 85 °C for different times periods showed that Nb M4 retained approximately 15% of its binding activity after incubation for 60 min at 85 °C. On the contrary, mAb 1E6 could only endure 10 min of heating at this temperature (Fig. 2B). Furthermore, the stability at the ELISA incubation temperature of 37 ℃ for various time periods was examined. As presented in Fig. 2C, Nb M4 retained approximately 80% of its binding activity after treatment for 60 min, whereas mAb 1E6 retained only about 55% of its binding activity. This is partly due to the ability of Nbs to refold from a thermally induced unfolded state to a native structure^55^. On the other hand, the extensive sequence and loop structure of CDR3, which augmented the binding area of Nbs to antigen, thus enabling it to tolerate high temperatures^56,57^. Meanwhile, the apparent melting temperature (T_m_) of both antibodies was determined using DSC analysis. As portrayed in Fig. 2D and E, the T_m_ of Nb M4 (65.1 ± 0.4 ℃) was higher than that of mAb 1E6 (T_m1_: 44.7 ± 0.2 °C, T_m2_: 54.9 ± 0.3 °C). Nbs have been reported to exhibit Tm values in the range of 47–85 ℃^58^, and the results from this study agree with this observation. Moreover, the T_m_ value of Nbs was positively correlated with their aliphatic index^57,59^. The aliphatic index of Nb M4 was 66.05 (https://web.expasy.org/protparam/), which might have contributed to its high-temperature tolerance. However, the thermal stability of Nb M4 was slightly lower than that of previously reported aflatoxicosis Nbs^45,60^. This difference could be due to Nb M4 being more hydrophobic and lacking additional disulfide bonds. Nevertheless, the results demonstrated that Nb M4 had a greater capacity to refold after denaturation and a higher ability to function after heat exposure than mAb 1E6, which is consistent with previous reports^61–63^. These findings highlight the potential of Nb M4 as a component for immunoassays operating under high-temperature conditions.

### Binding affinity of Nb M4

The efficacy of nanobodies is significantly influenced by their affinity for antigens. In this study, the binding affinity of Nb M4 to the AFM1-BSA conjugate was measured by the BLI system, which is a label-free biosensor that offers a direct and realistic depiction of molecular interactions via real-time kinetic measurements. The result established that Nb M4 binds to AFM1-BSA with a high affinity (*K*_D_ =2.5 nM) (Fig. 2F).

### Optimization of the icELISA for AFM1

To augment the icELISA sensitivity, the following experimental parameters were optimized: (i) optimal concentrations of AFM1-BSA and Nb M4; (ii) optimal AFM-1 solvent (methanol) concentration, and the reaction medium’s pH and ionic strength.

**Fig. 3:**
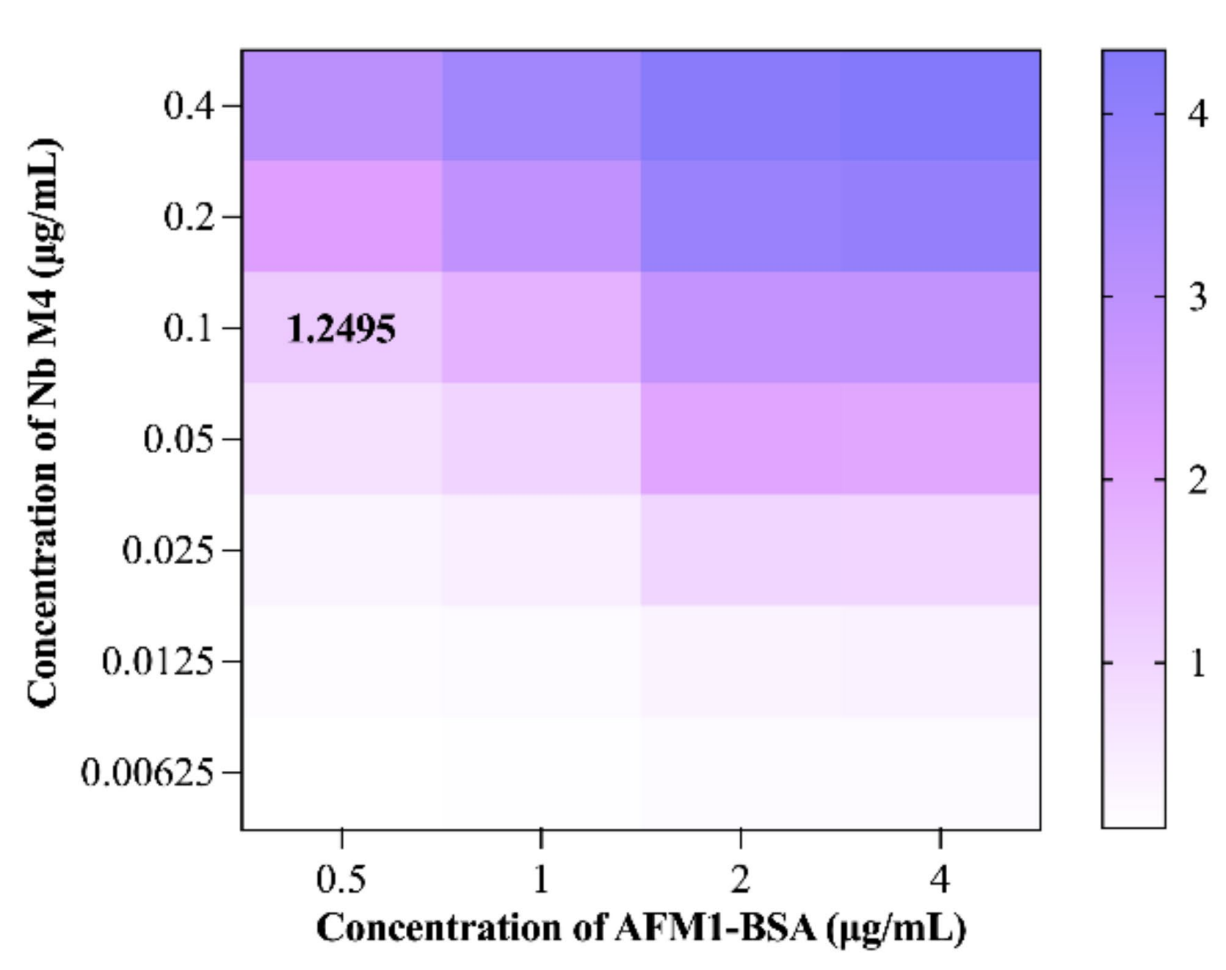
Optimal concentrations of Nb M4 and AFM1-BSA for icELISA development via checkerboard titration. The concentrations of Nb M4 and immobilized AFM1-BSA yielding a signal of approximately 1.0 were selected for icELISA development.

The optimal concentrations of AFM1-BSA and Nb M4 were initially determined via checkerboard titration (Fig. 3). In theory, the lower the antigen concentration used in a competitive reaction, the higher its sensitivity. Thus, the optimal concentrations of AFM-1BSA and Nb M4 were 0.5 µg/mL and 0.1 µg/mL, respectively. On this basis, the lowest IC_50_ and OD_max_ of approximately 1 were utilized as assessment standards to verify the other optimal parameters (including blocking conditions, methanol concentration, pH, and ionic strength).

**Fig. 4:**
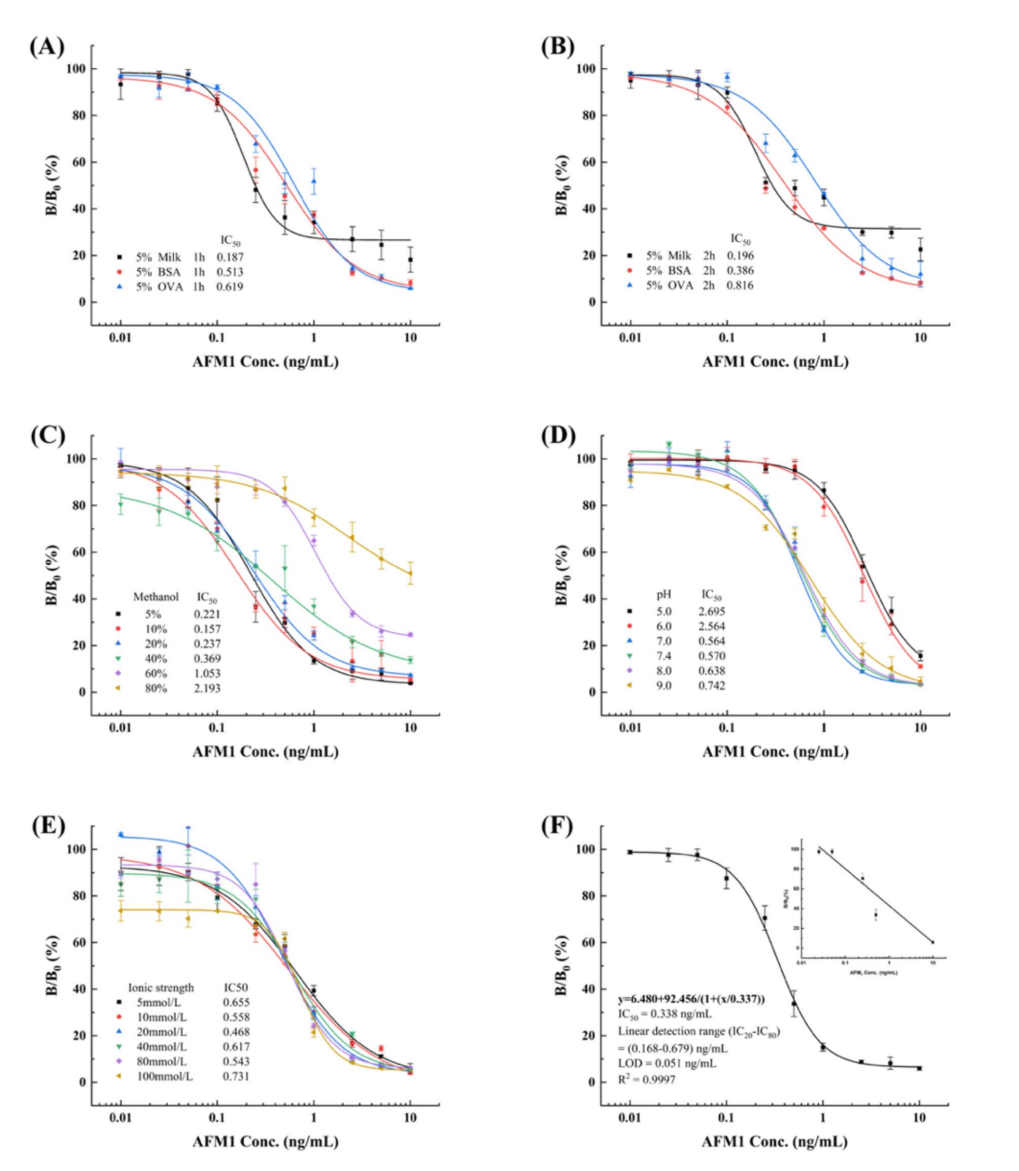
Optimal parameters of Nb M4-based icELISA. (**A**,**B**) Blocking conditions, (**C**) Methanol concentration, (**D**) pH, (**E**) ionic strength, (**F**) and standard curve of icELISA based on the optimal conditions (*n* = 3). The error bars represent the SD of independent measurements performed in triplicates.

Nonspecific adsorption can dramatically enhance the background signal, and blocking reagents are critical to reducing the nonspecific binding of Nbs^64^. Thus, 5% each of skim milk, BSA, and OVA were selected as blocking buffers, incubated at 37 ℃ for 1 h and 2 h, and evaluated (Fig. 4A and B). The lowest IC_50_ and a relatively low background were observed for 5% skim milk incubated for 1 h, which was therefore used in subsequent experiments. AFM1 is a lipophilic molecule that dissolves easily in methanol but not in water^65^. Nonetheless, high concentrations of methanol may affect antibody sensitivity. As depicted in Fig. 4C, the IC_50_ did not change significantly when the methanol concentration was < 40%, whereas it increased abruptly when the methanol concentration was > 60%. Therefore, 10% methanol (v/v) was selected for the subsequent experiments because a lower dilution would result in a higher final concentration for detection and appropriate maximum absorbance (Fig. 4C). Extreme pH values may disrupt the conformation of Nbs and impair their binding ability, which in turn affects the sensitivity of the immunoassay. The finding demonstrated that IC_50_ remained stable at pH 7.0–9.0, but it increased sharply in an acidic environment. This phenomenon can be explained by the isoelectric point of Nb M4 being 9.01. The lowest IC_50_ and OD_max_ of approximately 1 occurred at pH 7.4, which suggests that icELISA performed well at pH 7.4 (Fig. 4D). The addition of salt to the PBS stabilizes and improves the interaction of various analytes in the assay buffer^65^. Therefore, the effects of PBS solutions of different ionic strengths on reaction performance were evaluated (Fig. 4E). The IC_50_ value varied slightly from 0.468 ng/mL to 0.731 ng/mL, which showed the negative effect of ionic strength on the interaction between Nb M4 and AFM1. This phenomenon could be ascribed to the salting out of proteins, which could denature the structure of the antibodies and inhibit their activity owing to the high salt concentration in the assay buffer^66^. As PBS exhibited the lowest IC_50_, 20 mmol/L of this buffer was selected for subsequent experiments.

Under the optimized conditions, a standard curve for icELISA was plotted, as y = 6.480+92.456/[1+(x/0.337)] (R^2^ = 0.9997, *n* = 3) (Fig. 4F). The IC_50_ value was 0.338 ng/mL, LOD was 0.051 ng/mL, and with the linear range was 0.168–0.679 ng/mL. Previous studies have reported on idiotypic– Nbs generated from mAbs-immunized camelid used for AFM1 detection in dairy products^45,46,67^. In comparison, the IC_50_ of Nb M4 was 25-fold higher than that of VHH 4-1-1^67^ and slightly lower than that of VHH C4 reported by Cai et al. (2021)^46^. The detection limit and linear range of Nb M4 were comparable to that of VHH C4^46^. However, Nb M4 was much less sensitive than mAb-based similar approaches^68,69^ and commercially available kits (shown in Table 1). Nevertheless, Nb M4 complies with the limits set by several countries and organizations, which set a threshold of 0.5 µg/kg (500 ng/L)^70,71^. Furthermore, it demonstrates stability under thermal and organic solvent conditions, indicating its potential as a viable alternative immunoreagent for AFM1 detection assays.

**Table 1:** Detection performance of mAb-based commercial ELISA kit.

Assay format (manufacturer and catalog number)	Sensitivity, IC_50_; Linear Range, IC_20_-IC_80_; LOD, IC_10_	Cross-reaction rate (CR, %)	Recovery rate (%)	Precision (CV, %)
cELISA (CUSABIO, CSB-EL027236)	**LOD**: 0.03ng/mL **Linear range**: 0.03–2.43 ng/mL	AFM1: 100% AFB1: 12.9% AFB2: 1.4% AFG1: 1.9% AFG2: 0.3%	Milk: 90%±25% Milk powder: 100%±30% Yoghurt: 100%±30%	Intra-assay: CV < 10% Inter-assay: CV < 10%
cELISA (Cepham Life Sciences, CSD180420)	**LOD**: 0.02 ng/mL **Linear range**: 0.02–1.62 ng/mL	–	–	–
cELISA (Elabscience, E-TO-E007)	**LOD**: Milk: 0.1 ng/mL Milk powder: 0.15 ng/mL Urine: 0.5 ng/mL	AFM1:100%	Milk: 85%±15% Milk powder, Urine: 80%±15%	Intra-assay: CV < 8% Inter-assay: CV < 12%
cElisa (Abbexa, abx364886)	**LOD**: 0.05 ng/mL **Linear range**: 0.05–4.05 ng/mL	–	Milk: 85%±15% Milk powder, Urine: 80%±15%	–
cElisa (MyBioSource, MBS280162)	**LOD**: Milk: 0.1ng/mL Milk powder: 0.15ng/mL **Linear range**: 0.03–0.48 ng/mL	AFM1: 100%	Milk: 85%±15% Milk powder: 80%±15%	Intra-assay: CV < 8% Inter-assay: CV < 12%
cElisa (Abcam, ab302935)	**LOD**: Milk: 0.1 ng/mL Milk powder: 0.3 ng/mL Yoghurt: 0.3 ng/mL **Linear range**: 0.03–2.43 ng/mL	AFB1: 12.9% AFB2: 1.4% AFG1: 1.9% AFG2: 0.3%	Milk: 90%±25% Milk powder: 100%±30% Yoghurt: 100%±30%	Intra-assay: CV < 10% Inter-assay: CV < 10%

### Cross-reactivity

**Fig. 5:**
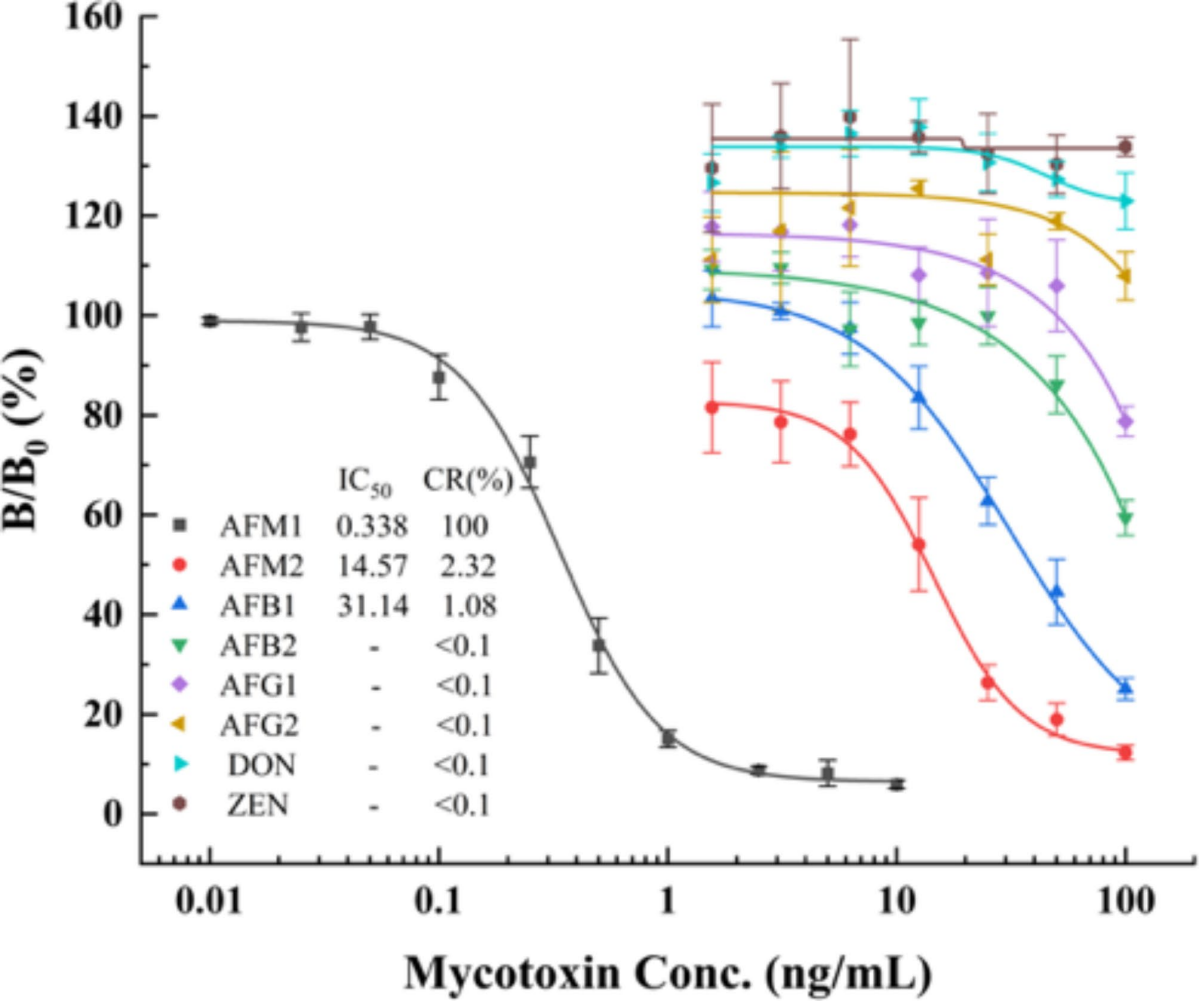
Cross-reactivity of the icELISA with different mycotoxins (i.e., AFM2, AFB1, AFB2, AFG1, AFG2, DON, and ZEN). The measurements are depicted as the mean ± SD of triplicates.

The specificity of Nb M4-based icELISA was evaluated by comparing the IC_50_ value of AFM1 with those of common mycotoxins, including AFM2, AFB1, AFB2, AFG1, AFG2, DON, and ZEN. As shown in Fig. 5, weak cross-reactions of 2.32% and 1.08% were observed for AFM2 and AFB1, respectively. Negligible cross-reactivity was observed for other compounds (< 0.1%). These findings indicate that the specificity of Nb M4-based icELISA is comparable to or better than that of some commercial kits (Table 1).

### Matrix effect

**Fig. 6:**
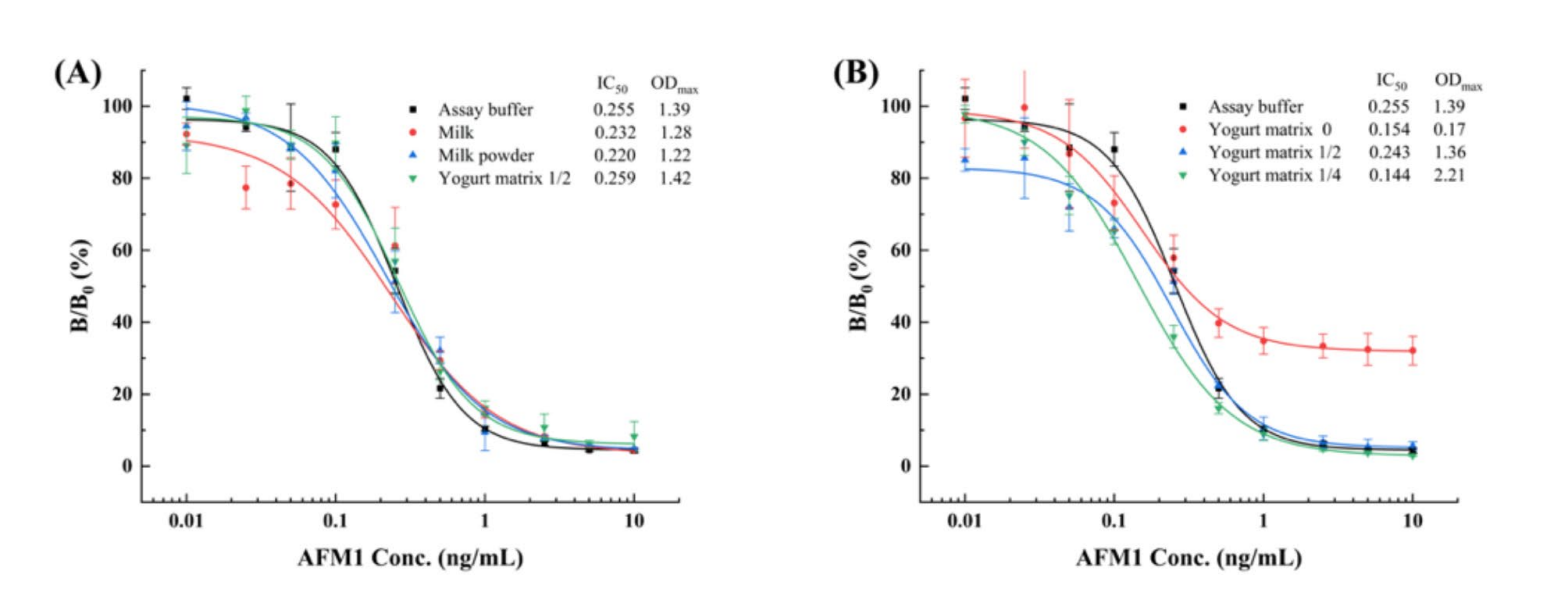
Standard curves of icELISA for detecting AFM1 in milk, yogurt, and milk powder samples after appropriate dilution. The measurements are depicted as the mean ± SD of triplicates.

The presence of matrix effects may reduce the accuracy of the assay and result in false positives or negatives^72^. Therefore, matrix interference is often eliminated by diluting the sample extract. However, high dilutions can lead to diminished sensitivity^73^. It was reported that the higher solubility and chemical stability of Nbs aid in minimizing minimize matrix effects^74^. To assess the potential matrix interference, milk, yogurt, and milk powder samples were selected as representative samples to ascertain the analytical performance of the method. These samples were procured from a local supermarket and were confirmed to be free of AFM1 using HPLC analysis. Three different dilutions of the sample matrix extract were utilized to construct the standard curves for comparison with the original one in 10% methanol-PBS buffer. The results showed that the competitive inhibition curve was not considerably altered when the AFM1 standard was diluted with milk or milk powder instead of 10% methanol-PBS, which implied that the matrix reaction could be ignored (Fig. 6A). The developed assay exhibited IC_50_ values of 0.232 ng/mL and 0.220 ng/mL in milk and milk powder, respectively, and linear detection ranges of 0.126–0.519 ng/mL and 0.074–0.734 ng/mL in milk and milk powder, respectively. However, yogurt had to be two-fold diluted with 1% BSA-PBS to eliminate matrix effects (Fig. 6B). After two-fold dilution, the IC_50_ was 0.243 ng/mL, with a linear detection range of 0.116–0.583 ng/mL.

### Analysis of the spiked samples and their validation

**Fig. 7:**
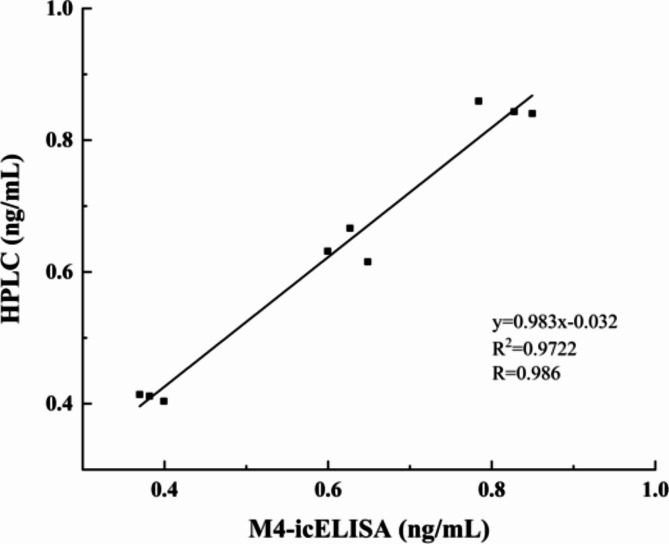
Correlation between the results of Nb M4-based icELISA and the HPLC for measuring AFM1 content in the spiked samples. The linear relationship equation of the two methods was y = 0.983x – 0.032x, R^2^ = 0.9722. The error bars represent the SD of independent measurements performed in triplicates.

To verify the accuracy of the Nb M4-based icELISA for sample analysis, spike-and-recovery experiments were performed, and the results were validated using the HPLC method described in GB 5009.24–2016 standard. Milk, yogurt, and milk powder samples spiked with three concentrations of AFM1 solution (0.4, 0.6, and 0.8 ng/mL) were prepared for the pretreatment. The intra-assay of Nb M4-based-icELISA exhibited an average recovery in the range of 94.5–111.33%, with a coefficient of variation of 1.62–4.30% (Table 2). The excellent correlation coefficient (R^2^ = 0.986) between the Nb M4-based icELISA and HPLC implied reliability and accuracy of the developed method for detecting AFM1 in dairy samples (Fig. 7).

**Table 2:** AFM1 recovery from milk, yogurt, and milk powder samples by Nb M4-based icELISA and HPLC (*n* = 3).

Sample	AFM_1_ spiked (ng/mL)	Nb ELISA			HPLC		
		mean ± SD (ng/mL)	Recovery (%)	CV (%)	mean ± SD (ng/mL)	Recovery (%)	CV (%)
Milk	0.4	0.391 ± 0.013	97.75	3.32	0.412 ± 0.015	103	3.64
	0.6	0.567 ± 0.029	94.5	5.11	0.632 ± 0.015	105.33	2.37
	0.8	0.829 ± 0.025	103.63	3.02	0.841 ± 0.027	105.13	3.21
Yogurt	0.4	0.398 ± 0.027	99.5	6.78	0.404 ± 0.015	101	3.71
	0.6	0.648 ± 0.010	108	1.54	0.616 ± 0.010	102.67	1.62
	0.8	0.827 ± 0.053	103.38	6.41	0.844 ± 0.017	105.5	2.01
Milk powder	0.4	0.380 ± 0.011	95	2.89	0.414 ± 0.012	103.5	2.90
	0.6	0.668 ± 0.055	111.33	8.23	0.666 ± 0.026	111	3.90
	0.8	0.814 ± 0.034	101.75	4.18	0.860 ± 0.037	107.5	4.30

### Real sample analysis

Twenty blind samples of milk (cow, camel, and goat), yogurt, and milk powder collected from local farms and supermarkets were simultaneously detected using icELISA and HPLC to confirm reliability. The concentration of AFM1 in all samples obtained from Nb M4-based icELISA was compared that from the HPLC, as presented in Table 3. A total of three samples were detected by HPLC as containing AFM1, while only one was detected by developed icELISA. This discrepancy was due to the residue amount of AFM1 in these two samples being below the detection limit of Nb M4, leading to non-detection, and rendering the developed method less sensitive than HPLC. Similar limitations have been reported in previous studies^45,46^. Therefore, its sensitivity needs to be improved in future studies. Nevertheless, these finding established that the Nb M4-based icELISA can be considered as an attractive and useful tool for the detection of AFM1 in dairy products.

**Table 3:** Comparison of AFM1 detected using nb M4-based icELISA and HPLC (*n* = 3).

Number	Sample	Nb ELISA (ng/mL ± SD )	HPLC (ng/mL ± SD)
1	Cow milk	ND^a^	0.029 ± 0.003
2		ND	ND
3		ND	ND
4		ND	ND
5		ND	ND
6		0.105 ± 0.046	0.110 ± 0.018
7		ND	ND
8		ND	0.088 ± 0.013
9	Camel milk	ND	ND
10		ND	ND
11		ND	ND
12	Goat milk	ND	ND
13		ND	ND
14		ND	ND
15	Yogurt	ND	ND
16		ND	ND
17		ND	ND
18	Milk powder	ND	ND
19		ND	ND
20		ND	ND

^a^ Each assay was conducted in triplicates on the same day. The results were expressed as the mean ± SD. ND = Not detected.

## Conclusion

In this study, the novel VH-like type anti-AFM1 Nb (Nb M4) obtained from the phage library in our previous work was utilized to assess the stability and affinity and develop an icELISA for AFM1 detection in dairy products. The higher thermal stability shown by Nb M4 over monoclonal antibody (mAb 1E6) renders it advantageous for immunoassay development, particularly for facilitating field testing in adverse environmental conditions. The developed method achieved a sensitive LOD of 0.051 ng/mL, with a linear range of 0.168–0.679 ng/mL. Although Nb M4 was not as sensitive as mAb-based ELISA kits, it still fulfills the requirement of the regulatory standard set by many countries. Additionally, Nb M4 exhibited outstanding specificity, negligible matrix effects, and high recovery rates, contributing to reduced false positives or improved accuracy in complex matrices. These findings support the feasibility of Nb M4-based icELISA for AFM1 detection in dairy products, while under the need for further research to enhance its sensitivity and validate its performance through direct comparisons with existing commercial kits.

## Electronic supplementary material

Below is the link to the electronic supplementary material.

Supplementary Material 1

## Data Availability

All data generated or analyzed during this study are included in this published article and its supplementary information files.
