# Supplementary material for: Nanobody-based indirect competitive ELISA for the detection of aflatoxin M1 in dairy products
Source: Sci Rep. 2025 Jan 4;15:785. doi: 10.1038/s41598-024-83869-4 (PMC11700155; doi:10.1038/s41598-024-83869-4)
Supplement: Supplementary file 1 — Supplementary Material 1 [file 41598_2024_83869_MOESM1_ESM.docx]

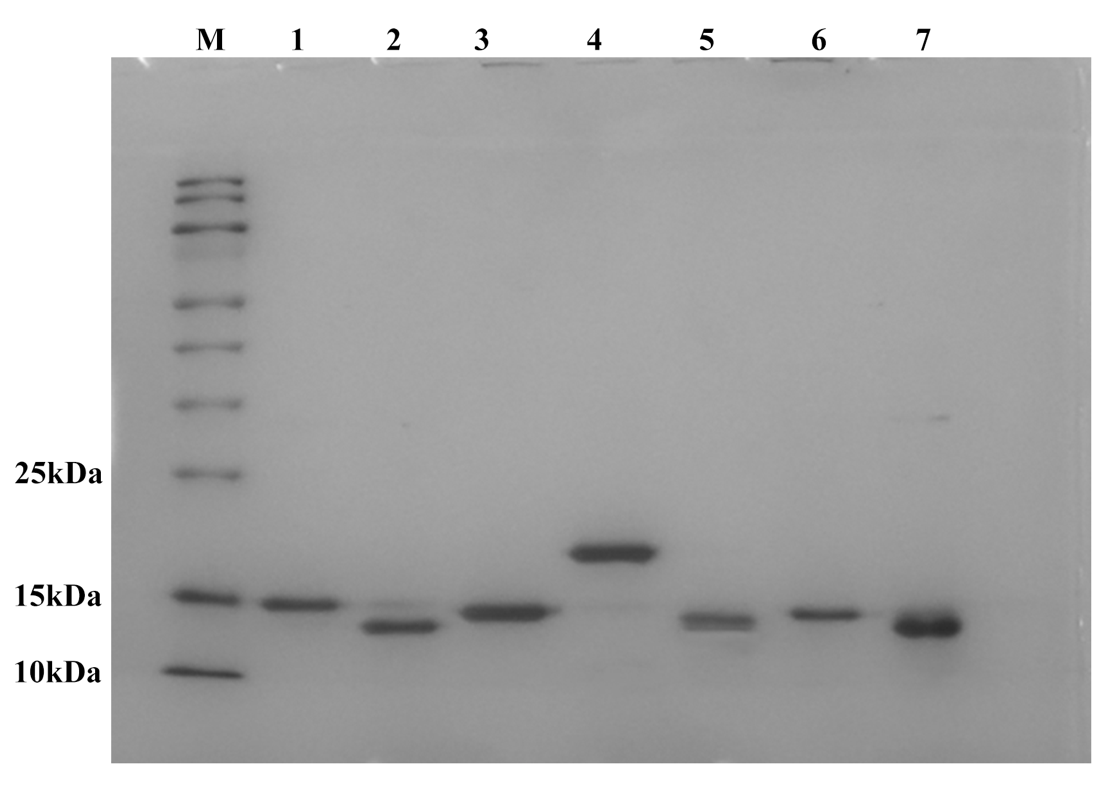


Figure S1. SDS-PAGE analysis of the purified Nbs. M: Marker, lanes 1–6: Nb M1–M6, and lane 7: Nb from another project, respectively.
